# Drug activity prediction using multiple-instance learning via joint instance and feature selection

**DOI:** 10.1186/1471-2105-14-S14-S16

**Published:** 2013-10-09

**Authors:** Zhendong Zhao, Gang Fu, Sheng Liu, Khaled M Elokely, Robert J Doerksen, Yixin Chen, Dawn E Wilkins

**Affiliations:** 1 Department of Computer and Information Science, School of Engineering, University of Mississippi, University, 38677, USA; 2 Department of Medicinal Chemistry, School of Pharmacy, University of Mississippi, University, 38677, USA; 3 Research Institute of Pharmaceutical Sciences, School of Pharmacy, University of Mississippi, University, 38677, USA

## Abstract

**Background:**

In drug discovery and development, it is crucial to determine which conformers (instances) of a given molecule are responsible for its observed biological activity and at the same time to recognize the most representative subset of features (molecular descriptors). Due to experimental difficulty in obtaining the bioactive conformers, computational approaches such as machine learning techniques are much needed. Multiple Instance Learning (MIL) is a machine learning method capable of tackling this type of problem. In the MIL framework, each instance is represented as a feature vector, which usually resides in a high-dimensional feature space. The high dimensionality may provide significant information for learning tasks, but at the same time it may also include a large number of irrelevant or redundant features that might negatively affect learning performance. Reducing the dimensionality of data will hence facilitate the classification task and improve the interpretability of the model.

**Results:**

In this work we propose a novel approach, named multiple instance learning via joint instance and feature selection. The iterative joint instance and feature selection is achieved using an instance-based feature mapping and 1-norm regularized optimization. The proposed approach was tested on four biological activity datasets.

**Conclusions:**

The empirical results demonstrate that the selected instances (prototype conformers) and features (pharmacophore fingerprints) have competitive discriminative power and the convergence of the selection process is also fast.

## Background

In drug discovery and development, researchers are not only interested in detecting which molecules are active, but also in determining which conformers of a given molecule are responsible for its observed biological activity. At the same time it is helpful to recognize the most representative subset of molecular descriptors to help in identifying desired properties to include in drug design. A molecule may adopt a wide range of conformers because of its structural flexibility. In order to understand the recognition mechanism between small flexible molecules and proteins, which is crucial in drug discovery and development, identification of the bioactive conformers becomes extremely important. However, the number of such structures is limited because of the experimental difficulty in obtaining the crystal structures, especially for transmembrane proteins, such as G protein-coupled receptors (GPCR) [1,2] and membrane transporters, even though the X-ray crystal structure of a ligand-protein complex is the most reliable way to obtain the bioactive conformer. Machine learning techniques are a good alternative to the traditional experimental approach. Machine learning is widely adopted in virtual screening to help prioritize candidate molecules for experimental molecule screening. Previously, Fu et al. [3] applied multiple-instance learning via embedded instance selection (MILES) to study the biological activity of several sets of molecules interacting with different receptor targets including glycogen synthase kinase-3 (GSK-3) [4], cannabinoid receptors (CBrs) [5], and P-glycoprotein (P-gp) [6]. MILES was shown to be highly competitive with classical quantitative structure-activity relationship (QSAR) approaches in terms of predictive abilities. During the work, Fu et al. observed that conformer and pharmacophore fingerprint features both reside in high dimensional spaces, which motivates us to extend the MILES framework with joint instance and feature selection. The selected prototype conformers and pharmacophore fingerprints may facilitate understanding of the interaction mechanism between small flexible molecules and proteins, and influence the design of a new molecule with desired properties, which is the goal in drug discovery and development.

### Multiple-instance learning

Multiple-instance learning is a variant of inductive machine learning. MIL was first introduced by Dietterich et al. [7] in the context of drug activity prediction. A multiple-instance problem involves a scenario as follows: A single example (bag) is a set of instances, a label is attached to the example, but not to the individual instances, each instance is represented by a feature vector respectively. One or more instances are responsible for the example's label, but labels for each instance are unknown. In the context of drug activity prediction, the observed activity (label) is associated with a molecule (bag), and the conformers (instances) of the molecule are responsible for its observed activity (label), but we do not know which conformers are bioactive (positive instances). Besides the drug activity prediction problem [7,8], MIL has been applied to a variety of challenging learning problems, including image categorization [9], natural scene classification [10], and text categorization [11].

Many techniques involving MIL can be found in the literature. We refer readers to the review of MIL [12]. MILES is a competitive and powerful multiple instance learning technique [13]. MILES assumes instances in the training set are representative, and maps each bag into the instance-embedded space represented by instances in the training set via a mapping function, which measures the similarity between a bag and a concept target (instance). Hence, MILES converts a MIL problem to a standard supervised learning problem, which can be solved by any standard supervised machine learning technique. However, the instance-embedded feature space often contains a large number of redundant or irrelevant features. Therefore 1-norm SVM is applied to eliminate redundant and irrelevant features to build a classifier.

### Feature selection

Feature selection is a machine learning technique of finding an "optimal" representative subset of features that has competitive discriminative power. Feature selection is much desired in scenarios where samples are represented in high-dimensional feature space, which hinders further analysis. In drug development, the most representative subset of prototype conformers and subset of molecule descriptors is crucial to facilitate understanding the principle of binding and designing a new molecule with the desired properties.

Methods of feature selection in supervised learning can be roughly categorized into three approaches [14,15]: filter, wrapper and embedded methods. A filter technique is a pre-processing method. The method ranks each feature individually and selects a subset of features; the selection is independent of the chosen learning predictor. A wrapper method uses some metric to search feature space to give a subset of features that produces the best prediction accuracy for a chosen learning machine, which is treated as a blackbox. An embedded method combines the feature subset selection and the learning algorithm together; the feature selection is performed during training. The learning procedure guides the feature selection, which tries to balance prediction performance and feature elimination. Some of the most successful feature selection algorithms belong to the embedded category. To the best of our knowledge, we are not aware of any embedded feature selection for MIL in the literature. Filter methods can be directly applied to MIL as it is classifier independent. For readers who are interested in more detail about feature selection in bioinformatics, please see [15-17].

A search technique for proposing new feature subsets is probably the most important component of a feature selection method. Search approaches in feature selection include [14,18]: exhaustive, best first, divide and conquer, greedy method, genetic algorithm, and simulated annealing. The greedy method, e.g. greedy forward selection or greedy backward selection, is the most popular search strategy, since it is typically robust to overfitness and has low variance [15].

Recently applying sparsity regularization in dimensionality reduction for feature selection has been widely investigated. For example, 1-norm regularization tends to give sparse solutions and was proposed to perform feature selection, e.g. 1-norm SVM [19,20].

### Our contribution

In this work, we propose a framework, multiple instance learning via joint instance and feature selection. The goal of the proposed approach is to produce a highly predictive model, and at the same time, provide the most representative subsets of prototype instances and features. Feature selection is embedded in the optimization procedure by 1-norm regularization, and zero-weighted features are eliminated iteratively, and a greedy backward elimination is also performed (optionally). First, standard MILES is applied on the training set. Instances in the training set are then classified and *k *top ranked positive and negative instances are selected respectively from the positive and negative training bags to compose a new data set which resides in the original feature space. Finally a new classifier is trained in the original feature space. In the framework, both classifiers are trained using 1-norm regularization, and sparse weight vectors are obtained. Those instances and features with zero weights are removed in the next iteration. The process repeats until there are no zero-weight features. Finally, an optional backward elimination is taken to find the best feature subset which gives the highest cross validation accuracy.

## Methods

In this section, in the first part we describe the proposed method: First we review instance-based mapping in MILES; then we describe instance classification and selection. Finally, we present our algorithm. In the second part we describe the method of generating data sets, data sets, and experimental setup.

For convenience of discussion in the second part, the same symbol notations as [13] are used in the paper. We denote the *i*th positive bag as $B_{i}^{+}$, where *i *= 1, . . . , *ℓ*^+^, and the *j*th instance in the bag as $x_{ij}^{+}$, where $j=1,\ldots ,n_{i}^{+}$. Similarly $B_{i}^{-}\left(i=1,\ldots ,\ell^{-}\right)$ and $x_{ij}^{-}\left(j=1,\ldots ,n_{i}^{-}\right)$ represent the *i*th negative bag and the *j*th instance in the negative bag, respectively. All instances are represented in the feature space  X. We line up instances in all bags and reindex them as **x***^k ^*(*k *= 1, . . . , *n*, where $n=\overset{\ell +}{\underset{i=1}{\sum }}n_{i}^{+}+\overset{\ell -}{\underset{i=1}{\sum }}n_{i}^{-}$). The superscripts + and *− *are omitted when the label of a bag does not matter.

### Instance-based feature mapping

Multiple-instance learning via embedded instance selection (MILES) converts a MIL problem to a standard supervised learning problem via instance-embedded feature mapping. MILES assumes each instance in the training set corresponds to a possible concept target $\left(C=\left\{x^{k}:k=\text{1},.\;.\;.,n\right\}\right)$. These concept targets (instances from the training bags) compose a new space, known as instance-embedded space, denoted as  F. A bag **B***_i _*is embedded in the space  F. The coordinates of **B***_i _*can be defined as

(1) $$m\left(B_{i}\right)={\left[s\left(x^{1},B_{i}\right),s\left(x^{2},B_{i}\right),\ldots ,s\left(x^{n},B_{i}\right)\right]}^{T},$$

where *s*(**x***^k ^*, **B***_i_*) can be a measure of similarity between the concept point and a bag **B***_i_*. In MILES, this is defined as

$$s(x^{k},B_{i})=\underset{i}{\text{max}}exp\text{(}-\frac{{||x_{ij},-,x^{k}||}^{2}}{\sigma^{2}}),$$

where *σ *is a parameter that has to be optimized during training of a classifier. In our drug activity prediction experiments, we use a simple formula,

(2) $$s(x^{\text{k}},B_{i})=\underset{i}{\min}\;d(x_{ij},x^{k}),$$

where *d *is a distance function, which measures the dissimilarity of two vectors. Hamming distance is used in our experiments since the pharmacophore fingerprint is a binary string.

After defining the instance-based feature mapping function, we can apply the function to a given training set to yield a matrix representation  M in  F:

(3) $$\begin{array}{l} \left[m_{1}^{+},\ldots ,m_{l^{+}}^{+},m_{1}^{-},\ldots ,m_{l^{-}}^{-}\right]\\ =m(B_{1}^{+}),\ldots ,m(B_{l^{+}}^{+}),m(B_{1}^{-}),\ldots ,m(B_{l^{-}}^{-})]\\ =\left[\begin{array}{ccc} s(x^{1},B_{1}^{+}) & \ldots & s(x^{1},B_{\ell^{-}}^{-})\\ s(x^{2},B_{1}^{+}) & \ldots & s(x^{2},B_{\ell^{-}}^{-})\\ \vdots & \ddots & \vdots \\ s(x^{n},B_{1}^{+}) & \ldots & s(x^{n},B_{\ell^{-}}^{-}) \end{array}\right], \end{array}$$

where each row represents a possible concept target in  F, each column represents a bag **B***_i_*.

For example, if a concept target **x***^k ^*achieves high similarity with some positive bags and low similarity with some negative bags via *s*(**x***^k^*, ·), where

$$s\left(x^{k},\cdot \right)=\left[m\left(B_{1}^{+}\right),\ldots ,m\left(B_{l^{+}}^{+}\right),m\left(B_{1}^{-}\right),\ldots ,m\left(B_{l^{-}}^{-}\right)\right],$$

the concept target **x***^k ^*is useful, because *s*(**x***^k^*, ·) provides some useful information to separate the positive bags and negative bags. These possible concept targets in  F usually are redundant and many of them are irrelevant. Feature selection is a necessary step to achieve high prediction performance. In MILES, instance (concept) selection is embedded in the classifier optimization via 1-norm regularization. An optimal classifier is learned and a subset of representative concepts is selected. Recursive instance selection is also possible.

After a learning iteration, an optimal sparse weight vector *w*^∗ ^and the corresponding intercept vector *b*^∗ ^are obtained. The magnitude of $w_{k}^{*}$ determines the contribution of the *k*th concept in  F to the learned classifier. The set of selected concepts is given as $\left\{x^{k}:k\in I\right\}$, where $I=\left\{k:\;|w_{k}^{*}|\;>0\right\}$, which are indices of non-zero weight entries in *w*^∗^. A concept target is named as a positive prototype if its corresponding weight is positive, or named as a negative prototype if its corresponding weight is negative. A bag **B***_i _*is classified as

(4) $$y=sign\left(\underset{k\in I}{\sum }w_{k}^{*}s\left(x^{k},B_{i}\right)+b^{*}\right),$$

which is computed solely using the positive and negative prototype concept targets. Void prototype concepts (see below) are irrelevant features which make no contribution to bag classification, because their corresponding weights are zero.

### Instance classification and selection

Instance classification in MIL is also important in some multiple instance learning problems. For example, finding the representative subset of molecule descriptors is as important as determining which molecules are active. We have to define a metric to label instances in a bag after a classifier for MIL is learned.

Labels of instances in a bag are unknown, and there is no ground truth. But we still can develop a formula to label instances after a multiple instance learner is trained: classifying instances in a bag according to their contributions to the bag classification.

Instances in a bag are classified into three categories: positive class, negative class, and void class, according to their contribution to $\sum_{k\in I}w_{k}^{*}s\left(x^{k},B_{i}\right).$ If an instance of a bag makes no contribution to classification of the bag, it is assigned to the void class. An instance in a bag is assigned to the positive class if its contribution is greater than some threshold; otherwise it is assigned to the negative class. The threshold has to be determined. Obviously, only instances of a bag which belong to the positive or negative class contribute to the classification of the bag. Instances of the void class can be safely dropped in equation (4).

**Figure 1:**
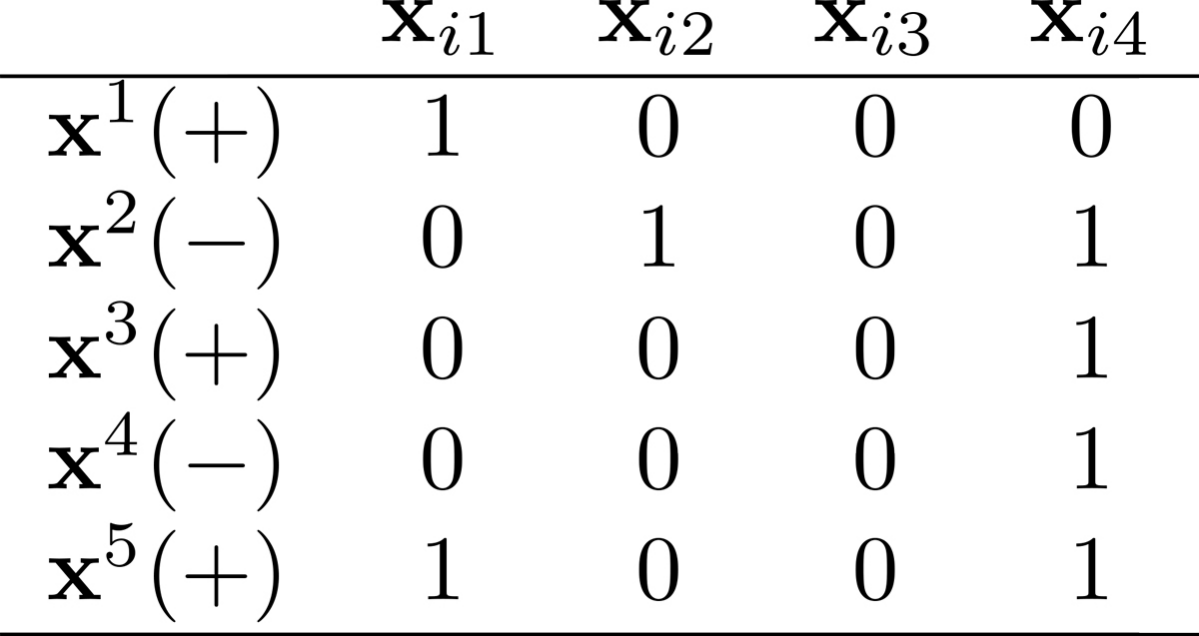
**A toy example for illustrating instance classification**.

For example, Figure 1 shows a bag **B***_i _*with four instances, **x***_ij_*, *j *= 1, 2, 3, 4, represented in columns. Five concept target points selected by a learning algorithm, I={1,2,3,4,5}, where **x**^1^, **x**^3^, **x**^5 ^are positive prototype points, **x**^2^, **x**^4 ^are negative prototype points, indicated by a plus or minus sign in parentheses, represented in rows. Each cell is filled with 1 or 0, where 1 (0) indicates the instance **x***_ij _*is (is not) the nearest neighbor to a concept target point k∈I.

We observe that a concept target has at least one instance that is its nearest neighbor. An instance of **B***_i _*could be the nearest neighbor of multiple concept target points, e.g., **x**_*i*1_ is the nearest neighbor of **x**^1 ^and **x**^5^. An instance can be the nearest neighbor of no **x***^k ^s*, e.g., **x**_*i*3_. **x**_*i*1_ can be classified as a positive instance since it is the nearest neighbor of positive concept target points only, **x**_*i*2_ can be classified as a negative instance since it is the nearest neighbor of only negative concept target points. **x**_*i*3_ can be classified as a void instance since it is not the nearest neighbor of any concept target points. **x**_*i*4_ has to be determined using a metric (see later), because it is the nearest neighbor of positive and negative concept points. The nearest neighbors of all concept targets together contribute to a bag classification.

Given a bag **B***_i_* with instances **x***_ij_*, *j *= 1, . . . , *n_i_*, we can define a minimal set of instances  U responsible for classifying **B***_i_* as

$$U=\{j^{*}:j^{*}=\underset{j}{\arg\;\min}\;d\left(x_{ij},x^{k}\right),\;k\in ℐ\},$$

which removes the instances of void class from the list, and the remaining instances $j^{*},j^{*}\in U$, of **B***_i_*, contribute to classifying **B***_i_*, e.g. U=1,2,4 for the above example (assuming *xi*_4_ is not classified as void class using (5)). Also given a k∈I, there could be multiple instances in **B***_i_* that are nearest neighbors of **x***^k ^*. The number of instances for **x***^k ^*is denoted as *m^k ^*. For the above example, *m^k ^*, *k *= 1, 2, 3, 4, 5 is 1, 2, 1, 1 and 2 respectively. Also an instance $x_{ij*}$, $j^{*}\in U$ could be one of the nearest neighbors for different **x **^k^k∈I. We introduce

$${ℐ}_{j*}=\{k:k\in ℐ,j^{*}=\underset{j}{\arg\;\min}\;d(x_{ij},x^{k})\},$$

for $j^{*}\in U$. And we define a new function,

(5) $$g\left(x_{ij^{*}}\right)=\underset{k\in I}{\sum }\frac{w_{k}^{*}s\left(x^{k},x_{ij^{*}}\right)}{m^{k}},$$

which defines the contribution of **x***_ij∗_* in a bag **B***_i_* to the classification of **B***_i_*. And the equation (4) is written as

$$y=sign\left(\underset{j^{*}\in U}{\sum }g\left(x_{ij^{*}}\right)+b^{*}\right).$$

Instances **x***_ij∗_* in a bag **B***_i_* can be classified using the criteria if *g*(**x***_ij∗_*) is greater than a threshold *δ*. The threshold *δ *is domain specific. In experiments of MILES, it was $-\frac{b^{*}}{\left|U\right|}$. In our experiments, we can avoid determining the threshold *δ *via ranking the decision values. Also it is arguable whether in equation (5), the factor of *m_k_* is necessary.

After the instances in the training set are classified, the instances and their predicted labels form a new learning problem and feature selection can be applied again. It is not difficult to apply the same learning algorithm on the instance data set, i.e., 1-norm regularization. An optimal sparse weight vector $w_{I}^{*}$ and the corresponding intercept vector $b_{I}^{*}$ are learned after the training process. The zero-weighted entries can be safely eliminated.

### An algorithmic view

**Figure 2:**
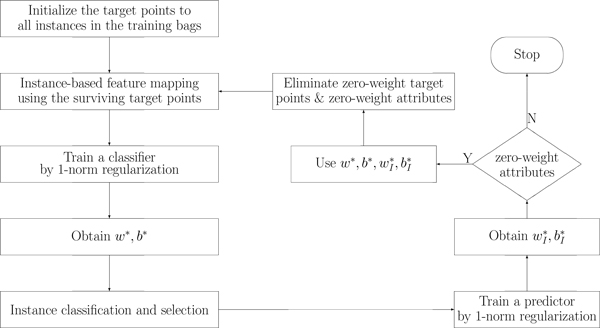
**Flowchart of MIL via joint instance and feature selection**.

We propose our approach based on the above discussion. The overall diagram of the method is illustrated in the Figure 2. The set of labeled bags is denoted as  B. The collection of instances from  B is denoted as $C=\;\left\{x^{k}:k=\text{1},\cdot \cdot \cdot ,n_{c}\right\}$, which resides in the feature space  X. The pseudo code in Algorithm 1 details the overall procedure to learn a classifier that iteratively selects the most representative instance and feature sets.

### Data sets

We tested our approach on four cheminformatics data sets [3]. Data set **I **includes all molecules exhibiting inhibitory activities for human glycogen synthase kinase-3 (GSK-3). Data sets **II **and **III **contain molecules modulating the intracellular activities of human Cannabinoid receptors (CBr). Since there are two identified CBr subtypes, CB1 and CB2, two different data sets were prepared to study the protein-small molecule interactions of the receptors separately. Some of the molecules which have reported binding affinities for both CB subtypes were included in both data sets **II **and **III**. Data set **IV **contained compounds which had been tested as substrates of P-glycoprotein (P-gp). The molecules in each data set were classified as positive or negative using cutoffs. For each data set, the molecules were partitioned into training and test set using a split around 3:1 respectively (see Table 1).

**Table 1:** Some statistics of data sets.

Data set	No. of molecules in training set		No. of molecules in test set		Total No. of molecules
		
	Positive	Negative	Positive	Negative	
**I**	199	188	67	70	524
**II**	191	210	62	74	537
**III**	247	131	60	57	495
**IV**	94	93	28	35	250

**Algorithm 1 **Pseudo code of MIL via joint instance and feature selection.

Input:

A set of labeled bags  B

Initial feature set  X

Output:

Selected Instances $C_{f}$

Selected Features $X_{f}$

1: *n ← *1, *$C_{0}=\emptyset$*, *$F_{0}=\emptyset$*, *$C_{1}=C$*, *$F_{1}=F$*

2: **repeat**

3:     **for **every bag $B_{i}=\left\{x_{ij}:j=\text{1},\cdot \cdot \cdot ,n_{i}\right\}$ in  B**do**

4:         **for **every instance **x***^k ^*in $C_{n}$**do**

5:             calculate $M_{ki}$ in (3) using (2)

6:         **end for**

7:     **end for**

8:     apply 1-norm regularization to the problem, get **w**^∗^, *b*^∗^, *Err_n_*

9:     $I_{n}\leftarrow \left\{k:|w_{k}^{*}|\;>\delta \right\}$

10:     $C_{n+1}\leftarrow \left\{x^{k}:k\in I\right\}$

11:     **for **every bag $B_{i}=\left\{x_{ij}:j=\text{1},\cdot \cdot \cdot ,n_{i}\right\}$ in  B**do**

12:         $U\leftarrow \{j^{*}:j^{*}=\arg\min_{j}d(x_{ij},x^{k}),\;k\in ℐ$

13:         *m_k_ ← *0 for every k∈I

14:         **X ***← *∅, **y ***← *∅

15:         **for **every *j*^∗ ^in  U**do**

16:             $I_{j}{}_{*}\leftarrow \{k:k\in ℐ,\;j^{*}=\arg\min_{j}d(x_{ij},x^{k})\}$

17:             *m_k_ ← m_k_* + 1 for every *k *in $I_{j*}$

18:         **end for**

19:         **for **every **x***_ij_* with *j*^∗ ^in  U**do**

20:             compute *g*(**x***_ij∗_*)

21:             ${z_{ij}}^{*}\leftarrow g\left({x_{ij}}_{*}\right)>-\frac{b^{*}}{\left|u\right|}$

22:         **end for**

23:         sort $\left\{g\left({x_{ij}}^{*}\right)*{z_{ij}}^{*},j^{*}\in U\right\}$ ascendingly to obtain corresponding indices of *g*(*x_ij∗_*)

24:         add the first *k *instances to **X **and corresponding labels to **y**

25:     **end for**

26:     apply 1-norm regularization to **X**, get $w_{I}^{*},\;b_{I}^{*}$

27:     **index ***← *index of $|w_{I}^{*}|>0$

28:     ${X_{n}}_{+\text{1}}\leftarrow X_{n}$(**index**)

29:     *n ← n *+ 1

30: **until **$X_{n}-{X_{n}}_{-\text{1}}=\emptyset$

31: **if **no backward elimination **then**                                                                                                          ⊳ Method 1: Natural stop

32:     *C_f _*= *C_n_*

33:     *F_f _*= *F_n_*

34: **else**                                                                                                                                  ⊳ Method 2: Backward elimination

35:     $n^{*}=\;arg\;min_{n}\;Err_{n}$

36:     *C_f _*= *C_n∗_*

37:     *F_f _*= *F_n∗_*

38: **end if**

The conformers of each molecule were generated using Macromodel module from Schrödinger Suite 2011. The average number of conformers for each molecule varies due to the molecules in the data sets having various conformational flexibilities (see Table 2). The pharmacophore fingerprints of each data sets were enumerated using Canvas 1.4 from Schrödinger Suite 2011. The pharmacophore fingerprint is a measure of molecular similarity based on 3D pharmacophoric shape. Each pharmacophore fingerprint is represented by a binary string, where each bit indicates the presence or absence of a match to a single pharmacophore model. The length of pharmacophore fingerprint for each data set is listed in Table 2.

**Table 2:** Number of conformers and length of pharmacophore fingerprint of data sets.

Data set	No. of conformers				Length of pharmacophore fingerprint
		
	Training		Test		
		
	Total	Average	Total	Average	
**I**	17429	44.6	5399	39.4	2979
**II**	35434	88.4	12333	90.7	14002
**III**	32528	86.1	9942	85.0	1542
**IV**	41960	224.4	10746	170.6	3467

### Data set generation

#### Generation of the pharmacophore hypothesis

We utilized Canvas version 1.5 [21-23] to convert the selected fingerprints into pharmacophore models. The pharmacophore fingerprints output was set to the bit code option for pharmacophore mapping. The bit code was then converted into a Phase [24-26] hypothesis (model).

#### 3D shape construction

The xyz-coordinates generated from the fingerprint-to-hypothesis step were then used to construct a 3D shape around the pharmacophoric elements. We used ROCS version 3.1.2 [27,28], to create a query (the pharmacophore model) from the xyz-coordinates, to align the query with the conformer generating the pharmacophore fingerprint, and to construct the 3D shape from the aligned query and its conformer.

#### Receptor grid preparation

We downloaded a set of 20 PDB structural files of GSK-3*β *from the RCSB Protein DataBank (PDB) [29] (PDB IDs: 1H8F, 109U, 1Q3D, 1Q3W, 1Q4L, 1Q5K, 1Q41, 1R0E, 1UV5, 2O5K, 2OW3, 3DU8, 3F7Z, 3F88, 3GB2, 3I4B, 3Q3B, 3ZRK, 3ZRL, and 3ZRM). We defined the protein and the complexed native ligand. A cubic box was constructed of dimension 8 Å around the ligand with a volume of an average of 6000 Å based on the ligand size in each case. A negative image with balanced extension towards the protein and solvent was created. The outer contour of the negative image was enabled for comparison with the docked poses [30].

#### Docking

We used Hybrid version 3.0.1 [31] to dock the conformers into all receptor grids. The selected conformers from the described approach were kept rigid during the docking step. The docked poses were compared with the native ligands and scored based on their shape fitting.

### Experimental setup

We implemented our approach in MATLAB, the 1-norm optimization problem was solved using IBM CPLEX LP solver [32]. The parameter *λ *was chosen using 10-fold cross validation (20-fold cross validation on Dataset IV). After a standard MILES learning step, two positive (negative) instances were selected from each positive (negative) training bag to form a new training data set in the original feature space. Each experiment was repeated five times. The run that gave the maximum cross validation accuracy was presented in the results that follow.

## Results and discussion

In this section, we present detailed results and discussion. Results of methods with and without backward elimination (denoted as Method 1 and Method 2, respectively) were both collected and presented below. The cross validation error, training error, test error, number of surviving instances and number of surviving features were collected and presented in Tables 3 to 7. The error rate *Err *is defined as the percent of falsely predicted bags, which equals one minus accuracy. We also present further analysis for data set **I **including a comparison with experimental evidence from co-crystallized binding conformations.

**Table 3:** Test error rates for each data set.

	**Error rate (%)**	R1	R2	R3	R4	R5	Average
Method 1	Data set **I**	18.25	14.60	17.52	10.95	19.71	16.20 ± 3.48 (11.82 ± 1.20)
	Data set **II**	18.38	16.18	17.65	19.85	15.44	17.50 ± 1.76 (16.91 ± 0.74)
	Data set **III**	16.24	14.53	15.38	16.24	17.95	16.07 ± 1.27 (15.38 ± 1.05)
	Data set **IV**	28.57	23.81	34.92	28.57	30.16	29.21 ± 3.98 (26.35 ± 0.87)

Method 2	Data set **I**	15.33	16.06	19.71	21.90	19.71	18.54 ± 2.76 (11.82 ± 1.20)
	Data set **II**	18.38	13.24	13.24	16.18	18.38	15.88 ± 2.58 (16.91 ± 0.74)
	Data set **III**	16.24	17.09	17.52	17.09	15.38	16.67 ± 0.85 (15.38 ± 1.05)
	Data set **IV**	37.30	34.92	26.98	28.57	36.51	32.86 ± 4.75 (26.35 ± 0.87)

R1-R5: from the first to the fifth repeat.

Note: numbers in parentheses are average error rates of the original MILES method.

### Results

We applied our approach on all four data sets. Tables 4 to 7 show the detailed results using our method with (without) backward elimination. The number of iterations in all four data sets is less than six, which suggests fast convergence of our method. In all of the tests, the first iteration always achieves the largest feature elimination. The error rates after joint instance and feature elimination are still competitive, and small subsets of representative instances and features are selected, which have good discriminative power. The error rates for each data set in five repeats are shown in Table 3. Method 1 (with backward elimination) performed slightly better than Method 2 (without backward elimination) in general. The numbers in parentheses represent the error rates of the original MILES method without instance and feature selection. The error rates for Method 1 and Method 2 are slightly higher than the original MILES method. However, our method eliminated most of the irrelevant and redundant features, and a small number of interesting instances were also selected at the same time. Each result is discussed below.

**Table 4:** Results of data set I

Iteration			0	1	2	3	4
Method 1	Error rate (%)	CV	19.23	14.10	11.67	-	-
		Training	3.88	7.24	5.43	-	-
		Test	13.14	13.14	17.52	-	-
	
	Number of instances		17249	153	80	-	-
	Number of features		2979	298	168	-	-

Method 2	Error rate (%)	CV	19.47	15.79	15.39	16.67	14.27
		Training	6.46	7.24	6.72	11.89	8.53
		Test	10.95	16.79	18.98	18.25	19.71
	
	Number of instances		17249	125	79	70	48
	Number of features		2979	284	158	117	111

Note: Iteration 0 is the original MILES method without instance and feature selection.

#### Data set I

Table 4 shows the results for data set **I **using the two methods. The feature elimination only took two iterations using Method 1. The initial sizes of instance-based and pharmacophore fingerprint feature space were 17249 and 2979, respectively. After applying Method 1, the final selected instances and fingerprints were 80 and 168, respectively. The first iteration eliminated the largest number of instances and features. The final accuracy was still competitive, although the error rate increased from 13.14% (the original MILES method, Iteration 0) to 17.52% (proposed method). Note that Iteration 0 is the original MILES method, which is without instance and feature selection. Without applying backward elimination (Method 2), the final error rate was higher (19.71% vs. 17.52%), however, the selected number of conformers and features are lower, 48 and 111, respectively.

#### Data set II

Data set **II **has the largest number of features and the second largest number of instances (see Table 2). Table 5 shows the results for data set **II**. Feature elimination worked very well and only took four iterations using Method 1. The final accuracy was slightly better, the error rate decreased from 16.18% to 15.44%. The initial sizes of instance-based and pharmacophore fingerprint feature space were 35434 and 14002, respectively. The final selected conformers and fingerprints were 23 and 19, respectively. The first iteration eliminated the largest number of instances and features. Method 2 also took four iterations and selected only one conformer and six pharmacophore fingerprints. The final error rate decreased slightly from 16.91% to 16.18%.

**Table 5:** Results of data set **II**.

Iteration			0	1	2	3	4	5
Method 1	Error rate (%)	CV	12.50	10.00	10.00	11.10	8.63	-
		Training	6.73	6.23	9.23	8.73	7.48	-
		Test	16.18	15.44	14.71	14.71	15.44	-
	
	Number of instances		35434	58	40	26	23	-
	Number of features		14002	106	43	35	19	-

Method 2	Error rate (%)	CV	12.50	8.75	10.00	10.00	12.50	12.50
		Training	4.99	6.98	11.97	11.47	13.72	13.72
		Test	16.91	19.85	14.71	15.81	16.18	16.18
	
	Number of instances		35434	62	37	10	7	1
	Number of features		14002	92	36	20	10	6

Note: Iteration 0 is the original MILES method without instance and feature selection.

#### Data set III

Again, the same tendency was observed for data set **III **(see Table 6). The final error rate was slightly better after feature elimination using Method 1 (17.09% vs. 16.24%). The initial number of instances and fingerprints were 32528 and 1542 respectively, the final selected instances and fingerprints were 22 and 36, respectively. It only took one iteration. Method 2 took three iterations and selected five conformers and two pharmacophore fingerprints. However, the error rate increased from 14.53% to 17.09%.

**Table 6:** Results of data set **III**.

Iteration			0	1	2	3
Method 1	Error rate (%)	CV	5.26	5.26	-	-
		Training	6.08	6.88		
		Test	17.09	16.24		
	
	Number of instances		32528	22	-	-
	Number of features		1542	36	-	-

Method 2	Error rate (%)	CV	5.33	6.69	9.21	7.89
		Training	5.56	5.82	8.73	8.73
		Test	14.53	15.39	17.09	17.09
	
	Number of instances		32528	48	14	5
	Number of features		1542	55	8	2

Note: Iteration 0 is the original MILES method without instance and feature selection.

#### Data set IV

Data set **IV **has the largest number of instances, and the average number of instances per molecule was 224, which indicates molecules in the data set are highly flexible. The data set size was also smaller, compared with the other data sets. Table 7 shows the results for data set **IV**. The final error rate was slightly improved after feature elimination using Method 1, going from 25.40% to 23.81%. The selected number of instances was decreased from 41960 to 8, and the selected number of fingerprints were decreased from 3467 to 27. Method 2 only took two iterations and selected 13 conformers and 54 pharmacophore fingerprints. The final error rate slightly increased from 26.98% to 28.57%.

**Table 7:** Results of data set **IV**.

Iteration			0	1	2	3	4	5
Method 1	Error rate (%)	CV	30.00	22.22	22.22	22.22	22.22	22.22
		Training	17.65	22.46	22.46	24.60	24.06	25.13
		Test	25.40	20.64	23.81	20.64	22.22	23.81
	
	Number of instances		41960	40	15	8	8	8
	Number of features		3467	142	56	50	48	27

Method 2	Error rate (%)	CV	22.22	22.22	22.22	-	-	-
		Training	19.79	25.67	25.13	-	-	-
		Test	26.98	30.16	28.57	-	-	-
	
	Number of instances		41960	27	13	-	-	-
	Number of features		3467	90	54	-	-	-

Note: Iteration 0 is the original MILES method without instance and feature selection.

### Interpretation of results for dataset I

**Figure 3:**
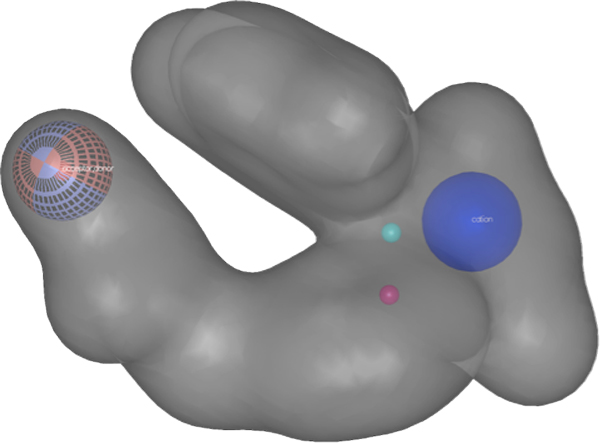
**The four pharmacophoric elements selected by our approach: blue solid sphere (positive ionizable group), cyan and red small spheres (hydrophobes), and mixed red and cyan meshed sphere (hydrogen bond acceptor/donor)**. The 3D shape is constructed to provide the main skeleton of possible hits.

**Figure 4:**
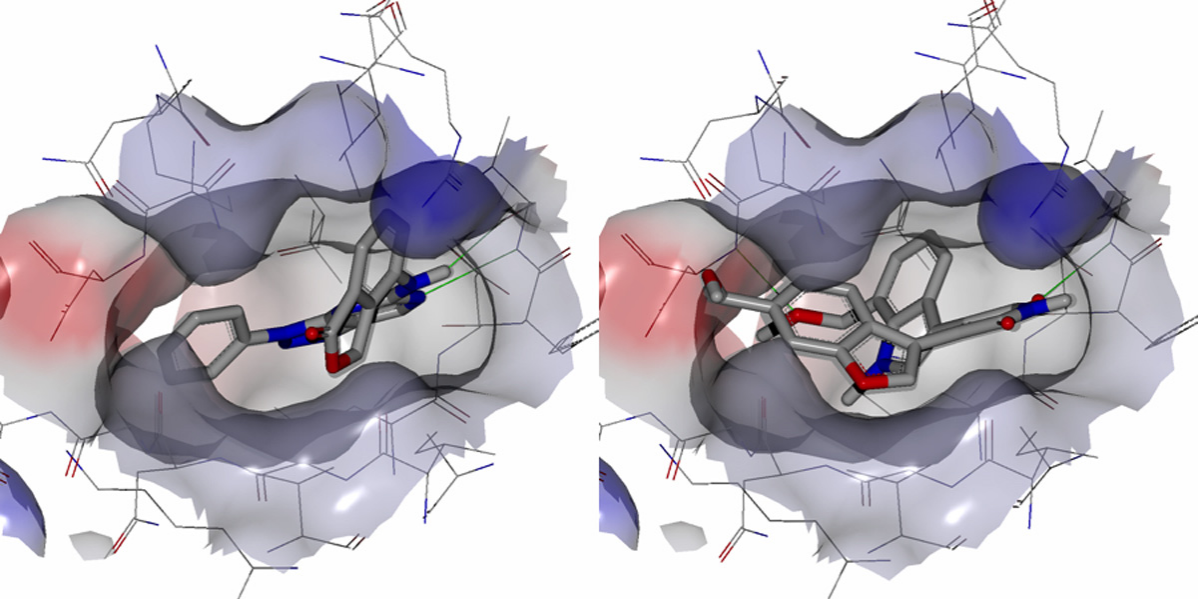
**Conformers fitting inside the receptor grid**. AK-4 (left) and DPAP-4 (right) show a perfect fit with the 1Q4L.pdb grid.

**Figure 5:**
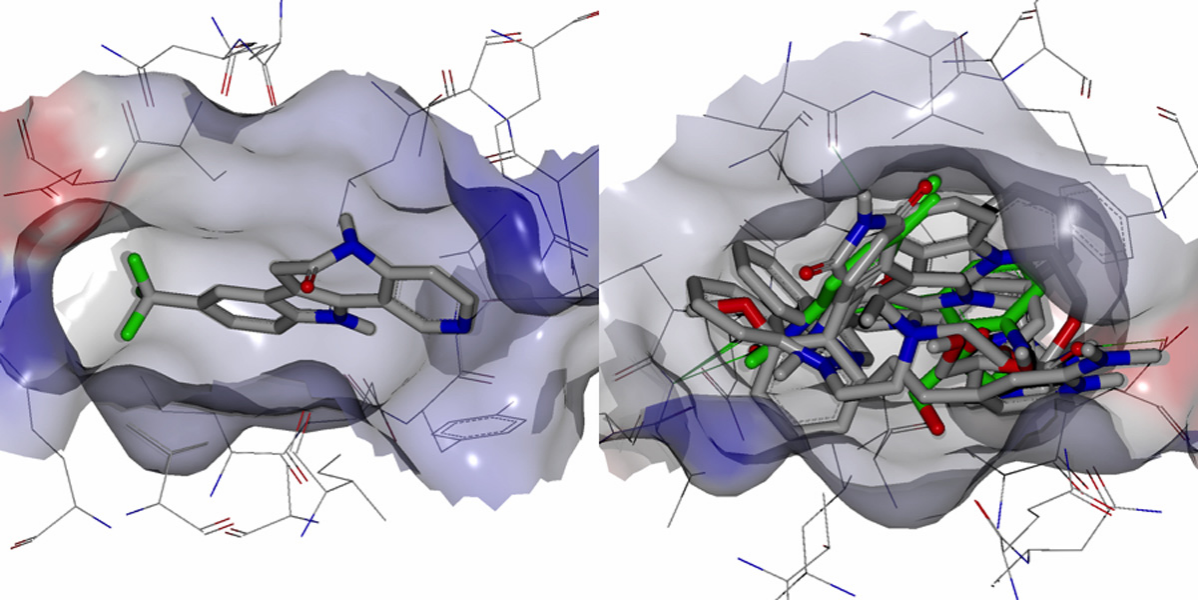
**Different conformers fit well into different grids**. Only one fit perfectly into1Q3W.pdb grid (left) while four conformers fit perfectly into 1R0E.pdb grid (right).

**Figure 6:**
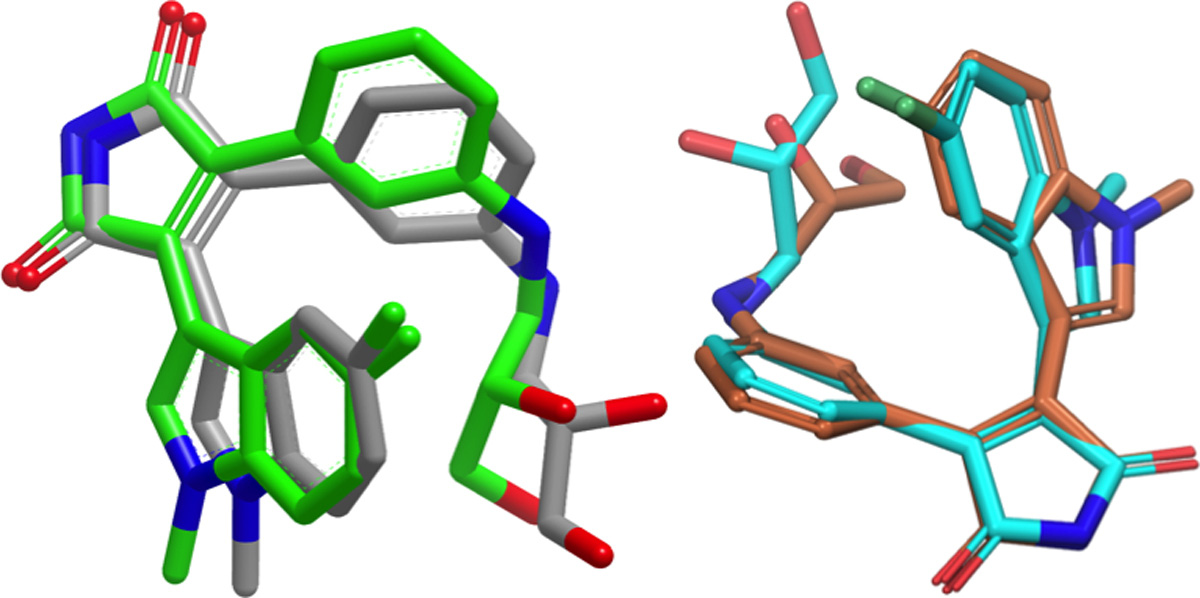
**Comparison of native ligand conformation and one of a very closely related molecule**. Receptor grid based alignment (left), native ligand in green, selected conformer in grey. Pharmacophore based alignment (right), native ligand cyan, selected conformer in orange. Perfect alignments are found in both cases, repesenting the ability of this approach to find the bioactive conformer.

The results of data set **I **using Method 1 led to selection of 80 conformers and more than 168 pharmacophore fingerprints. All the selected conformers came from molecules that have less than 12 nM inhibitory activity. Due to the large number of selected conformers, we analyzed a representative set consisting of the 17 with the best biological activities. For interpretation of the vast quantity of data, we considered only the pharmacophore fingerprint which matched the greatest number of conformers, and call it P1. P1 consists of four pharmacophore features: a hydrogen bond acceptor/donor, two hydrophobes and a positively ionizable group. The 3D shape surrounding these pharmacophoric elements was generated using ROCKS in OpenEye to provide the general structural features of the pharmacophore model. The 17 selected conformers fit well with the 3D shape and with the four pharmacophoric elements. To understand how the pharmacophore models correlate with inhibition of the protein, we compared the selected conformers with receptor grid shapes (as defined in OpenEye). When using rigid docking to the 20 receptor grids, some of the grids could not dock any of the conformers. The selected conformers are clustered into eight groups based on their shape complementarity to the receptor grids (PDB IDs: 1Q3W, 1Q4L, 1R0E, 1UV5, 2OW3, F88, 3GB2, 3ZRM). In order not to bias the conformer pose into the receptor, we considered the rigid fitting approach in Hybrid version 3.0.1, and then compared the poses with that of the native ligand in each receptor. The conformers fit well inside the receptors and showed high correlation with native ligands (Figures 3 to 6).

We can conclude that:

1. The approach we are presenting is capable of predicting active ligands and the correct poses or in other words, the bioactive conformers of each ligand.

2. The pharmacophore models fit well with the receptor shapes.

3. This approach will simplify the drug design process. We can depend on the described technique to find the suitable bioactive conformers for each hit instead of using an ensemble of receptors or multiple receptors (such a large amount of structural data often is not available). In the current case, one pharmacophore model showed a good fit with eight different GSK-3*β *structures, and can be used in virtual screening instead of having to use the eight protein structures.

## Conclusions

In this paper, we have proposed a new method which extends a multiple-instance learning (MIL) framework, multiple-instance learning via embedded instance selection (MILES), with joint instance and feature selection. The method formulates the classification problem as an optimization problem using 1-norm regularization. The embedded feature elimination is automatically achieved because of the intrinsic property of 1-norm regularization that it tends to give a sparsity-favoring solution. The method identifies a very small subset of representative instances and features effectively and quickly through iterative instance and feature eliminations. The experimental results on four data sets show that the iterative elimination process typically completes in just a few iterations and eliminates the largest number of instances and features in the first iteration. Compared with MILES, which does not perform feature elimination, our method achieves a competitive classification accuracy and selects very small discriminative subsets of instances and features at the same time.

In the future, we will continue to experimentally investigate biological significance of the selected prototype conformers and pharmacophore fingerprints to validate our method. We also would like to investigate instance classification further, which plays a critical role in our method. Due to limited publicly available data sets, we have to find and collect more data sets to evaluate our proposed method more extensively.

## Competing interests

The authors declare that they have no competing interest.

## Authors' contributions

ZZ, YC, DEW, and RJD planned the research. ZZ prepared the computer code and ran the calculations. ZZ, GF, LS, KE, YC, DEW, and RJD analyzed the results. ZZ wrote the manuscript with assistance from YC, DEW and RJD. All authors carefully reviewed the manuscript before submission.

## Declarations

The authors declare that funding for publication of the article was sponsored by NSF under award numbers EPS 0903787.

This article has been published as part of *BMC Bioinformatics *Volume 14 Supplement 14, 2013: Proceedings of the Tenth Annual MCBIOS Conference. Discovery in a sea of data. The full contents of the supplement are available online at http://www.biomedcentral.com/bmcbioinformatics/supplements/14/S14.

## References

[B1] Fanelli F De Benedetti P Computational modeling approaches to structure-function analysis of G protein-coupled receptors Chemical Reviews 2005 105 3297 3351 10.1021/cr000095n 16159154

[B2] Klabunde T Hessler G Drug design strategies for targeting G-protein-coupled receptors Chem-BioChem 2002 3 928 944 10.1002/1439-7633(20021004)3:10<928::AID-CBIC928>3.0.CO;2-5 12362358

[B3] Fu G Nan X Liu H Patel R Daga P Chen Y Wilkins D Doerksen R Implementation of multiple-instance learning in drug activity prediction BMC Bioinformatics 2012 13 Suppl 15 S3 10.1186/1471-2105-13-S15-S3 23046442 PMC3439725

[B4] Cohen P Goedert M GSK3 inhibitors: Development and therapeutic potential Nat Rev Drug Discov 2004 3 479 487 10.1038/nrd1415 15173837

[B5] Pavlopoulos S Thakur G Nikas S Makriyannis A Cannabinoid receptors as therapeutic targets Curr Pharm Des 2006 12 1751 1769 10.2174/138161206776873743 16712486

[B6] Matheny C Lamb M Brouwer K Pollack G Pharmacokinetic and pharmacodynamic implications of P-glycoprotein modulation Pharmacotherapy 2001 21 778 796 10.1592/phco.21.9.778.34558 11444575

[B7] Dietterich T Lathrop R Lozano-Perez T Solving the multiple instance problem with axis-parallel rectangles Artif Intell 1997 89 31 71 10.1016/S0004-3702(96)00034-3

[B8] Maron O Ratan A Multiple-Instance Learning for Natural Scene Classification Proceedings of the 15th International Conference on Machine Learning: 24-27 July 1998 1998 Madison 341 349

[B9] Chen Y Wang JZ Image Categorization by Learning and Reasoning with Regions J Mach Learn Res 2004 5 913 939

[B10] Maron O Lozano-Perez T A framework for multiple-instance learning Adv Neur 1998 10 570 576

[B11] Andrews S Tsochantaridis I Hofmann T Support Vector Machines for Multiple-Instance Learning Adv Neur 2003 15 561 568

[B12] Foulds JR Frank E A review of multi-instance learning assumptions Knowledge Eng Review 2010 25 1 25 10.1017/S026988890999035X

[B13] Chen Y Bi J Wang J MILES: Multiple-instance learning via embedded instance selection IEEE Trans Pattern Anal Mach Intell 2006 28 1931 1947 17108368 10.1109/TPAMI.2006.248

[B14] Molina L Belanche L Nebot A Feature selection algorithms: a survey and experimental evaluation Data Mining, 2002. ICDM 2003. Proceedings. 2002 IEEE International Conference 2002 306 313

[B15] Guyon I Elisseeff A An introduction to variable and feature selection J. Mach. Learn. Res 2003 3 1157 1182

[B16] Ma S Huang J Penalized feature selection and classification in bioinformatics Briefings in Bioinformatics 2008 9 5 392 403 10.1093/bib/bbn027 18562478 PMC2733190

[B17] Saeys Y Inza In Larrañaga P A review of feature selection techniques in bioinformatics Bioinformatics 2007 23 19 2507 2517 10.1093/bioinformatics/btm344 17720704

[B18] Kohavi R John GH Wrappers for Feature Subset Selection ARTIFICIAL INTELLIGENCE 1997 97 273 324 10.1016/S0004-3702(97)00043-X

[B19] Bradley P Mangasarian OL Feature Selection via Concave Minimization and Support Vector Machines Machine Learning Proceedings of the Fifteenth International Conference(ICML '98 1998 Morgan Kaufmann 82 90

[B20] Nan X Wang N Gong P Zhang C Chen Y Wilkins D Biomarker discovery using 1-norm regularization for multiclass earthworm microarray gene expression data Neurocomput 2012 92 36 43

[B21] Duan J Dixon SL Lowrie JF Sherman W Analysis and comparison of 2D fingerprints: Insights into database screening performance using eight fingerprint methods Journal of Molecular Graphics and Modelling 2010 29 2 157 170 10.1016/j.jmgm.2010.05.008 20579912

[B22] Sastry M Lowrie JF Dixon SL Sherman W Large-Scale Systematic Analysis of 2D Fingerprint Methods and Parameters to Improve Virtual Screening Enrichments J. Chem. Inf. Model 2010 50 5 771 784 10.1021/ci100062n 20450209

[B23] Canvas, version 1.5, Schrödinger, LLC, New York, NY, 2012 http://www.schrodinger.com

[B24] Dixon S Smondyrev A Knoll E Rao S Shaw D Friesner R PHASE: a new engine for pharmacophore perception, 3D QSAR model development, and 3D database screening: 1. Methodology and preliminary results J Comput Aided Mol Des 2006 20 647 671 10.1007/s10822-006-9087-6 17124629

[B25] Dixon SL Smondyrev AM Rao SN PHASE: A Novel Approach to Pharmacophore Modeling and 3D Database Searching Chemical Biology & Drug Design 2006 67 370 372 10.1111/j.1747-0285.2006.00384.x 16784462

[B26] Phase, version 3.4, Schrödinger, LLC, New York, NY, 2012 http://www.schrodinger.com

[B27] Swann SL Brown SP Muchmore SW Patel H Merta P Locklear J Hajduk PJ A Unified, Probabilistic Framework for Structure- and Ligand-Based Virtual Screening Journal of Medicinal Chemistry 2011 54 1223 1232 10.1021/jm1013677 21309579

[B28] ROCS version 3.1.2. OpenEye Scientific Software, Santa Fe, NM http://www.eyesopen.com

[B29] The Research Collaboratory for Structural Bioinformatics PDB http://www.rcsb.org/pdb/

[B30] FRED version 3.0.1. OpenEye Scientific Software, Santa Fe, NM http://www.eyesopen.com

[B31] HYBRID version 3.0.1. OpenEye Scientific Software, Santa Fe, NM http://www.eyesopen.com

[B32] ILOG CPLEX Optimization Studio. IBM Academic Initiative http://www-03.ibm.com/ibm/university/academic/pub/page/ban_ilog_programming

